# The effectiveness of high-intensity interval training on body composition, cardiorespiratory fitness, and cardiovascular risk factors in children: A protocol for a systematic review

**DOI:** 10.1097/MD.0000000000019233

**Published:** 2020-02-21

**Authors:** Montserrat Solera-Martínez, Ana Díez-Fernández, Alberto González-García, Ismael Manzanares-Domínguez, Vicente Martínez-Vizcaíno, Diana P. Pozuelo-Carrascosa

**Affiliations:** a Universidad de Castilla-La Mancha, Health and Social Research Center; b Universidad de Castilla-La Mancha, Facultad de Enfermería, Cuenca, Spain.; c Universidad Autónoma de Chile, Facultad de Ciencias de la Salud, Talca, Chile.; d Universidad de Castilla-La Mancha, Facultad de Fisioterapia y Enfermería, Toledo, Spain.

**Keywords:** body composition, cardiometabolic health, cardiorespiratory fitness, children, high-intensity interval training

## Abstract

**Background:**

No previous systematic review has examined the effect of high-intensity interval training (HIIT) interventions on body composition, cardiometabolic risk factors and cardiorespiratory fitness (CRF) in healthy schoolchildren from 5 to 12 years old.

**Methods:**

This study will be conducted by following the guideline of the preferred reporting items for systematic review and meta-analysis protocols. An electronic search in MEDLINE (via PubMed), EMBASE (via Scopus), SPORTDiscus, Cochrane Library and Web of Science databases of all dates from inception will be conducted. We will include randomized controlled trials aimed to assess the effectiveness of HIIT to improve cardiometabolic risk factors, body composition, and CRF in children. Two authors will perform the study selection and data collection; disagreements will be solved by a third reviewer. The methodological quality of studies will be assessed by the Cochrane Collaboration's tool for assessing risk of bias (RoB2). Data analysis and synthesis will be performed by Comprehensive Meta-analysis Software and StataSE software, version 15.

**Conclusion:**

The results should be disseminated through publication in a peer-reviewed journal. Since the data used in systematic reviews of this type will be extracted exclusively from published studies, approval form and ethics committee will not be required.

## 1 Introduction

The prevalence of overweight and obesity has increased in recent decades and remains high among children and adolescents.^[1]^ Worse levels of cardiovascular risk factors in childhood are related with early atherosclerosis and cardiac pathology^[2]^; thus, children with greater number of cardiovascular disease risk factors may develop atherosclerosis in adulthood at an accelerated rate.^[3]^

Traditionally, continuous aerobic exercise in children, characterized as moderate intensity exercise without rest intervals, has been the most common type of exercise recommended to improve body composition, cardiorespiratory fitness (CRF) and overall health-related parameters^[4–6]^ (eg, blood pressure, insulin resistance, and lipid profile). However, the training duration of continuous aerobic exercise must be sufficiently long to induce the desirable effect and thus, long exercise duration leads to poor exercise adherence due to monotony.^[7]^

Recently, interest in higher intensity interval training (HIIT) research has increased. HIIT has been identified as a time-efficient type of exercise to promote health benefits and improve fitness.

Numerous meta-analyses have reported that HIIT leads to improvements on body composition^[8]^ and CRF^[9–11]^ in healthy adolescents and adults. Besides, HIIT may improve physiological adaptations and may induce greater health benefits.^[5]^ Greater reduction in total body mass, fat mass, trunk fat, and fasting plasma insulin levels has been reported in several intermittent and continuous exercise comparison studies in obese adults.^[7,12]^

Particularly, there is limited evidence related to outcomes of intermittent exercise. It is discussed that it may be more appropriate for children because their physical activity patterns are naturally intermittent.^[13]^ However, HIIT performed in healthy children is significantly less investigated than in obese children, adolescents, adults or chronic disease patients; so, it remains uncertain whether HIIT is the best form of exercise for children to maintain or improve cardiometabolic health.

Therefore, the aim of this study protocol is to provide a clear, standardized and transparent methodology for performing a systematic review and meta-analysis aimed to assess the effect of HIIT programs versus non-training Control Groups (CGs) on body composition, cardiometabolic risk factors and CRF in healthy children.

## 2 Methods

### 2.1 Protocol and registration

This systematic review and meta-analysis protocol is based on the preferred reporting items for systematic reviews and meta-analyses (PRISMA) statement^[14]^ and following the recommendations of the Cochrane Handbook for Systematic Reviews^[15]^ of Interventions.

The meta-analysis study was registered with the PROSPERO International Prospective Register of Systematic Reviews Database (CRD42018093432).

### 2.2 Search strategy

Relevant randomized controlled trials (RCTs) will be searched in MEDLINE (via PubMed), EMBASE (via Scopus), SPORTDiscus, Cochrane Library, and Web of Science databases RCTs with language restriction in Spanish and English. In addition, the literature search will be complemented by manual screening references included in articles that are considered eligible for the systematic review. Study records will then be managed using the Mendeley reference manager. The search strategy will include free text terms, combining Boolean operators from the relevant concepts presented in Table 1.

**Table 1:**
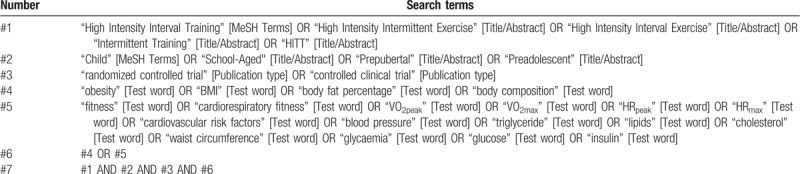
Full-search strategy for MEDLINE database.

### 2.3 Eligibility criteria

#### 2.3.1 Study design

All relevant RCTs assessing HIIT to improve body composition, cardiometabolic risk factors and/or CRF in children, in which the CG did not receive any PA intervention will be included. Observational, prospective cohort studies systematic reviews, and RCTs performed with adolescents or adults will be excluded.

#### 2.3.2 Participants

The studies must include healthy schoolchildren aged 5 to 12 years.

#### 2.3.3 Interventions

The included studies must involve interventions of HIIT, prescribing high intensity exercise if

(1) the intensity was >= 90% peak oxygen consumption^[16]^ (VO_2peak_);

(2) had an intensity that was >= 100% maximal aerobic speed^[17]^; and/or ensuring that the participant's heart rate (HR) was >= 90% of their peak HR (HR_peak_).^[18,19]^ The studies also could be included if HIIT is combined with nutritional intervention in the CG.

#### 2.3.4 Outcomes

The primary outcomes will be the following: changes in body composition assessed with body mass index (BMI) and body fat percentage; changes in the levels of some of cardiometabolic risk factors such as systolic and diastolic blood pressure, total cholesterol, low-density lipoprotein cholesterol (LDL-c), high-density lipoprotein cholesterol (HDL-c), glucose, triglyceride and changes in the CRF assessed as maximal oxygen consumption (VO_2max_), VO_2peak_, maximal HR (HR_max_) and HR_peak._

### 2.4 Selection of studies and data extraction

**Figure 1:**
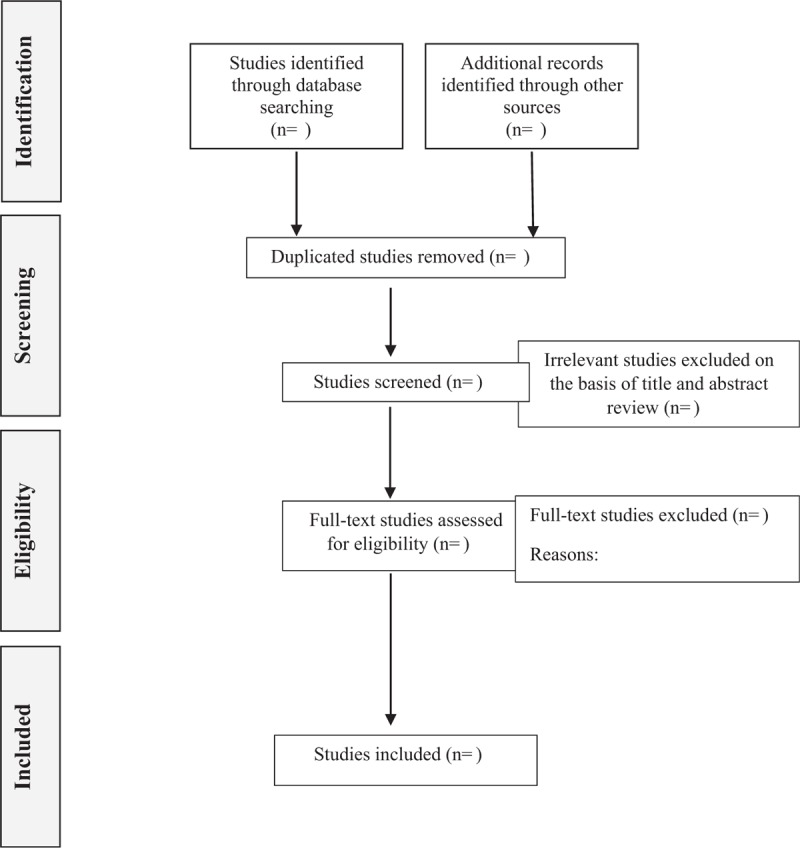
The study selection will be conducted according to the PRISMA statement. PRISMA = preferred reporting items for systematic reviews and meta-analyses.

After excluding duplicated records, 2 researchers will independently evaluate the titles and abstracts of the retrieved articles to identify eligible studies for the systematic review. Abstracts that meet inclusion criteria or that do not provide enough information regarding the inclusion/exclusion criteria will then be evaluated through a full-text reading. Then, 2 researchers will examine the included and excluded studies to verify the reason for each decision. Inconsistencies between these 2 researchers regarding selection will be resolved by a third researcher, who will make the final decision. The selection process of eligible articles is shown in Figure 1.

The data extraction will be carried out by 2 independent reviewers in accordance with the standardized sheet recommended by the Cochrane Handbook of Systematic Reviews of Interventions. The following information will be extracted from the included studies:

(1) first author's name,
(2) year of publication,
(3) characteristics of participants (number, age, weight status),
(4) HIIT and CG protocols (type, duration, modality, and intensity), and
(5) results of the means and standard deviations of body composition, cardiometabolic risk factors and CRF variables from pre- to post-intervention between groups (HIIT vs CG).

### 2.5 Missing data dealing with

When we encounter missing or unclear data, we will try to contact corresponding authors for additional information.

### 2.6 Risk of bias assessment

Two independent researchers will be blinded to the authors, titles and years of publication of the included studies to evaluate the risk of bias of each included study. The risk of bias assessment will be conducted using the RoB 2.0 tool developed by the Cochrane Collaboration.^[20]^ This version includes 5 domains: bias arising from the randomization process, bias due to deviations from intended interventions, bias due to missing outcome data, bias in measurement of the outcome, and bias in selection of the reported result. Each domain is scored as high risk, some concerns or low risk. A sixth domain, overall bias, will have a low risk result if the rest of the domains are of low risk, some concerns if there are some domains assessed with some concerns; and high risk if there are 1 or more domains with high risk of bias.

### 2.7 Data analysis and synthesis

Meta-analyses will be conducted to determine the effect of HIIT on body composition, cardiometabolic risk factors, and CRF variables, in comparison to nontraining CGs. For studies that include both non-training CGs and continuous moderate intensity exercise comparison groups, only the CG data (without physical activity intervention) will be included in the meta-analysis.

The effect size (ES) of each study will be calculated as the standardized mean differences in the variables from pre- to post-intervention between groups (HIIT vs CG) using random-effects model^[21]^ (DerSimonian-Laird approach). Finally, the ES of all studies included will be combined to estimate an overall summary ES, with a 95% confidence interval and a random-effects model.

The heterogeneity results across studies will be evaluated using the *I*^2^ statistic, and the following values will be used for its interpretation: 0% to 30% “not important” heterogeneity; >30% to 50% “moderate” heterogeneity; >50% to 80% “substantial” heterogeneity, and >80% to 100% “considerable” heterogeneity. The corresponding *P*-values will be also considered.^[15]^

For each pooled ES, we will be conducted a sensitivity analysis by removing the studies one by one to assess the robustness of the summary estimates and to detect whether any study accounted for a large proportion of heterogeneity. Finally, visual inspection of funnel plots and the Egger test will be conducted to detect publication bias.^[22]^

A lineal meta-regression analysis will be used to explore whether covariates could be associated with the magnitude of the effect and could explain the observed statistical heterogeneity.^[15]^

In addition, subgroup analyses will be performed, if it is possible, to assess whether the weight status influences the overall ES. All analyses will be perform using Comprehensive Meta-analysis Software (2nd version, Biostat, Englewood, NJ) and StataSE software, version 15 (StataCorp, College Station, TX).

## 3 Discussion

To the best of our knowledge, this is the first systematic review and meta-analysis protocol to examine the effect of HIIT interventions on body composition, cardiometabolic risk factors and CRF in healthy schoolchildren aged 5 to 12 years. HIIT could be considered a more effective and time-efficient intervention for improving cardiometabolic health maskers in children compared with a non-exercise CG.

Several meta-analyses have assessed the effectiveness of physical activity interventions to improve cardiometabolic risk factors^[23]^ and CRF^[24]^ in children. However, the majority of meta-analysis aimed to assess the effectiveness of HIIT to improve the cardiometabolic health have been conducted in adolescents or adults,^[25]^ which justifies the need for this study.

Furthermore, the results of this study will provide rigorous summary evidence about the effects of HIIT interventions on body composition, cardiometabolic risks factors such as blood pressure, BMI, HDL-c, LDL-c, triglycerides, glucose, total cholesterol, and CRF in healthy children.

## 4 Limitations

Different issues should be acknowledged as potential limitations: information and publication bias, insufficient methodological quality, scarce of statistical analysis, inclusion of articles in English and Spanish exclusively and inadequate reporting of methods and results. To try to avoid these possible limitations, we will take into account potential risks of bias for each study and we will follow the existing guidelines included in the PRISMA^[14]^ and the Cochrane Collaboration Handbook.^[15]^

## 5 Ethics and dissemination

Due to the nature of systematic review and meta-analysis, patients’ informed consent and ethic approval will not be required. The results obtained of this study could be particularly useful because they will provide rigorous evidence regarding the effect of HIIT interventions to improve the children's cardiometabolic health.

## Author contributions

**Conceptualization:** Solera-Martínez, M, Pozuelo-Carrascosa DP.

**Data curation:** Solera-Martínez M, Díez-Fernández A, Pozuelo-Carrascosa DP.

**Formal analysis:** Solera-Martínez M.

**Investigation:** Solera-Martínez M, Manzanares-Domínguez I, Pozuelo-Carrascosa DP.

**Methodology:** Solera-Martínez M, Díez-Fernández A, González-García A, Pozuelo-Carrascosa DP.

**Project administration:** Solera-Martínez M, Pozuelo-Carrascosa DP.

**Supervision:** Martínez-Vizcaíno V.

**Writing – original draft:** Solera-Martínez M, Pozuelo-Carrascosa DP.
